# The Discrete and Continuous Brain: From Decisions to Movement—And Back Again

**DOI:** 10.1162/neco_a_01102

**Published:** 2018-09-01

**Authors:** Thomas Parr, Karl J. Friston

**Affiliations:** Wellcome Trust Centre for Neuroimaging, Institute of Neurology, University College London, WC1N 3BG, U.K. thomas.parr.12@ucl.ac.uk; Wellcome Trust Centre for Neuroimaging, Institute of Neurology, University College London, WC1N 3BG, U.K. k.friston@ucl.ac.uk

## Abstract

To act upon the world, creatures must change continuous variables such as muscle length or chemical concentration. In contrast, decision making is an inherently discrete process, involving the selection among alternative courses of action. In this article, we consider the interface between the discrete and continuous processes that translate our decisions into movement in a Newtonian world—and how movement informs our decisions. We do so by appealing to active inference, with a special focus on the oculomotor system. Within this exemplar system, we argue that the superior colliculus is well placed to act as a discrete-continuous interface. Interestingly, when the neuronal computations within the superior colliculus are formulated in terms of active inference, we find that many aspects of its neuroanatomy emerge from the computations it must perform in this role.

## 1 Introduction

The nervous system faces a dual challenge in shaping behavior. To induce changes in the external world, it is necessary to contract muscles or secrete chemicals. Such processes necessarily involve the manipulation of continuous variables—muscle length or chemical concentration. In addition, animals must make decisions. To do so, they must entertain several different possible courses of action, or policies. Ultimately, they must select one of these actions or policies that are necessarily discrete. We draw on recent work that considers the interactions between the neuronal processing of discrete and continuous quantities (Friston, Parr, & de Vries, 2017). To make this more concrete, we focus on the oculomotor system. Sampling the visual world entails decisions about where to look and the implementation of these decisions by contraction of the extraocular muscles.

We use perceptual inference performed by the networks supporting eye movements as a way of motivating and illustrating the theoretical challenge we want to address. However, the treatment we offer generalizes to any system that involves the physical implementation of categorical decisions. The ideas presented in this article complement previous treatments of cognitive time (VanRullen & Koch, 2003), including the notion of a perceptual moment (Allport, 1968; Shallice, 1964; Stroud, 1967) and the suggestion that brain oscillations act as discrete clocks to support this type of computation (Buschman & Miller, 2009, 2010). They also resonate with recent developments in machine learning (Linderman et al., 2017) and some of the problems faced in modern robotics (Cowan & Walker, 2013; Schaal, 2006). In short, the coupling of categorical decision making and dynamic perception (and motor control) raises some deep questions about the temporal scheduling of perception (and action).

Oscillatory rhythms in measured brain activity have been linked to cyclical perceptual processes (Buzsaki, 2006), with theta and alpha cycles as the most popular hypothesized units of perceptual time (VanRullen, 2016). In endorsement of this, the timing of processing relative to the phase of certain oscillations appears to be important (Buzsáki, 2005). While there is some controversy concerning the frequency of the perceptual clock, an advantage to focusing on the oculomotor system is that we can evade this issue. The frequency of spontaneous saccadic sampling is around 4 Hz, allowing us to commit to a theta rhythm. Conveniently, this is the frequency often associated with attentional and central executive (decision) functions (Chelazzi, Miller, Duncan, & Desimone, 1993; Duncan, Ward, & Shapiro, 1994; Hanslmayr, Volberg, Wimber, Dalal, & Greenlee, 2013; Landau & Fries, 2012; VanRullen, 2013), as opposed to sensory processes associated with faster frequencies (Drewes & VanRullen, 2011; Dugué, Marque, & VanRullen, 2011; Ergenoglu et al., 2004; van Dijk, Schoffelen, Oostenveld, & Jensen, 2008).

The oculomotor system is a distributed network that includes brain stem, cortical, and subcortical regions (Parr & Friston, 2017a). An important point of contact between the cortical oculomotor networks and those in the brain stem is the superior colliculus (Raybourn & Keller, 1977), found in the midbrain. This structure receives a dual input from the cortex (Fries, 1984) and the basal ganglia (Hikosaka & Wurtz, 1983) and provides an important input to the brain stem oculomotor nuclei. In the following, we argue that the connectivity implied by active inference is consistent with a role for the superior colliculus as an interface between the discrete and continuous processing of the oculomotor system.

This article is organized as follows. In section 2, we review the principles of active inference, their application to discrete and continuous state spaces, and the relationship between the two. In section 3, we relate the computational anatomy implied by active inference to the neuroanatomy of oculomotion. In section 4, we illustrate oculomotor behavior, and its neural correlates, through simulation. Section 5 presents the discussion, and section 6 concludes.

## 2 Active Inference

### 2.1 Principles of Active Inference

Active inference is the process of minimizing variational free energy through action and perception (Friston, Samothrakis, & Montague, 2012; Friston, Daunizeau, Kilner, & Kiebel, 2010). The imperative to maintain a low free energy stems from the self-evidencing (Hohwy, 2016) nature of living systems. If an organism samples sensory input (e.g., blood pressure, pH, and temperature) compatible with life, this constitutes evidence for its existence as the sort of thing it is. It is intuitively sensible that living creatures should act so that they experience such sensations.

In more formal terms, the adaptive fitness of a creature's phenotypic state or trait “just is” the probability of a creature being in that state. This probability can be treated as model evidence by associating the creature with a model and its sensory exchange with the world with phenotypic or characteristic states. By casting exchange with the world in terms of a random dynamical system, it is fairly straightforward to show that the states that constitute the creature must, on average, increase model evidence; hence self-evidencing. (See Friston, 2013, for details.)

Active inference formalizes this notion by casting behavior as free-energy-minimizing or self-evidencing processes. Perception follows from recognizing or inferring the causes of sensory input by optimizing probabilistic representations with respect to free energy. Crucially, these causes include our own actions. This means action selection can be treated as “planning is inference” (Attias, 2003; Botvinick & Toussaint, 2012). In short, perceptual inference underlies action, active sensing informs perception, and both serve to minimize free energy or maximize model evidence.

Mathematically, the relationship between free energy and model evidence can be expressed through Jensen's inequality:

$$\underset{\log\;\mathrm{evidence}}{\underbrace{\ln p(y)}}=\underset{\mathrm{Jensen}\text{"}s\;\mathrm{inequality}}{\underbrace{\ln\left[E_{q(\theta )}\frac{p(y,\theta )}{q(\theta )}\right]\geq E_{q(\theta )}\left[\ln\frac{p(y,\theta )}{q(\theta )}\right]}}=\underset{\mathrm{negative}\;\mathrm{free}\;\mathrm{energy}}{-F}.$$

This equation shows that the evidence associated with observations, y (i.e., sensory data) for a given system is always greater than or equal to the negative free energy. The free energy is a function of two things. The first is a generative model, p(y,θ), that describes how sensory data are generated from latent (“hidden”) variables, θ. The second is an arbitrary (“recognition”) distribution, q(θ), that becomes an approximate posterior when the free energy is minimized (Dayan, Hinton, Neal, & Zemel, 1995; Friston, 2009):

$$q\left(\theta \right)\approx p\left(\theta |y\right)\Leftrightarrow \frac{\delta F}{\delta q(\theta )}=0.$$

To ensure that free energy minimization is tractable, this distribution is often assumed to factorize into several marginal distributions. This is referred to as a mean-field approximation (Feynman, 1998). Although originally introduced to solve problems in physics, it appears to be consistent with the anatomical separation of processing streams in the brain (Friston & Buzsáki, 2016). For example, the division of visual processing into dorsal (“where”) and ventral (“what”) streams (Ungerleider & Haxby, 1994) suggests that the brain factorizes beliefs about these variables, implementing something like a mean-field approximation. The approximation takes the form

$$q\left(\theta \right)=\prod_{i}q(\theta_{i}).$$

If we substitute this into the expression for the free energy above and then take the variational derivative with respect to these marginals, we find the optimal value for this distribution (Beal, 2003):

(1) $$\left.\begin{array}{c} q\left(\theta_{i}\right)\propto \exp\left(E_{q(\theta \backslash i)}\left[\ln p(y,\theta )\right]\right)\\ q\left(\theta \backslash i\right)=\prod_{j\neq i}q(\theta_{j}) \end{array}\right\}\Leftrightarrow \frac{\delta F}{\delta q(\theta_{i})}=0.$$

This solution is common to variational inference about discrete and continuous data and is key to understanding the rest of this article. To apply it in each of these domains, we must consider that the form of the probabilistic generative model is encoded by p(y,θ). In the remainder of this section, we show how this simple equation can be applied to inference in discrete and continuous state-space models, allowing us to address the general problem of oculomotor control in terms of neuronal (variational) message passing (Dauwels, 2007; Winn, 2004).

**Figure 1:**
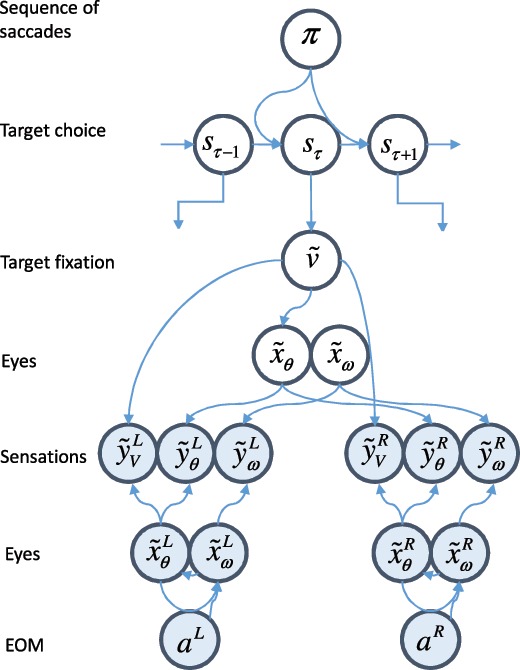
From decisions to eye movements. This figure shows the form of a generative model (unfilled circles) that generates predictions about visual and proprioceptive data, $\tilde{y}$, from the policy, π, discrete hidden states, $s_{\tau }$, continuous hidden causes, $\tilde{v}$, and continuous hidden states, $\tilde{x}$, that the brain believes are in play. The filled circles indicate the generative process that describes how data are generated by physical processes in the external world. Superscripts refer to the left or right eye. Subscripts indicate modalities (θ= angular position; ω= angular velocity; V indicates vision). Note that action, a, of the extraocular muscles (EOM) induces changes in angular velocity (i.e., accelerations) and that the generative model assumes only one gaze direction despite the fact that the generative process allows for each eye to move independently. This is based on the generative model and process in Parr and Friston (2018), but extends it with the addition of a discrete level. Figure 2 provides more detail on the mathematical specification of these types of models.

This inference problem is illustrated as a Bayesian network in Figure 1. We first address inferences about the upper part of this network that selects target fixation locations. We then consider how these inform beliefs about sensory data and generate changes in the external world.

### 2.2 Deciding Where to Look

**Figure 2:**
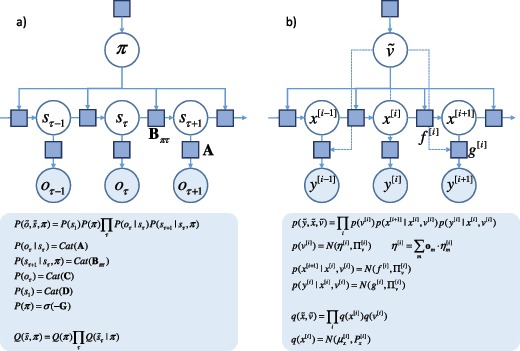
Discrete and continuous generative models. (a) This graphic shows a Markov decision process in factor graph form (Loeliger et al., 2007). Blue squares indicate probability distributions (factors of the generative distribution). Below, these factors are expressed in terms of probability matrices. “Cat” denotes a categorical distribution. The implicit mean-field factorization of the approximate distribution, Q, is shown. (b) This shows the equivalent structure for a continuous state-space model. Heuristically, we can think of these (generative) models as an algorithm that generates data. This would involve drawing variables from their prior distributions (e.g., $\pi ,s_{1},\tilde{v})$, and using these variables to sample dependent variables (e.g., $s_{2},\tilde{x})$ from conditional distributions. Finally, the data ($\tilde{o},\tilde{y})$ can then be generated from the likelihood. In short, a generative model is just a probabilistic specification of how data are caused. Note that the prior mean for $\tilde{v}$ is derived from the outcomes of the discrete model. See the main text and Figure 1 for an explanation of the variables.

The first computations we address are those that mediate decisions about where to look. Decisions involve the selection of one from several alternatives—in our example, saccadic targets. As such, (Markov) decision processes are a natural form for the generative model because they are defined on discrete state spaces (FitzGerald, Schwartenbeck, Moutoussis, Dolan, & Friston, 2015; Friston, FitzGerald, Rigoli, Schwartenbeck, O'Doherty, et al., 2016; Friston, FitzGerald, Rigoli, Schwartenbeck, & Pezzulo, 2017; Friston et al., 2015; Friston et al., 2013; Friston et al., 2014; Friston, Lin, et al., 2017; Friston, Parr, et al., 2017; Friston, Rosch, Parr, Price, & Bowman, 2017; Mirza, Adams, Mathys, & Friston, 2016; Parr & Friston, 2017c, 2017d; Schwartenbeck, FitzGerald, Mathys, Dolan, Wurst, et al., 2015). Our latent variables are the hidden states of the world, s, that represent fixation locations. Each policy, π (course of action), that can be pursued is represented as a competing model. As time progresses, hidden states undergo transitions that depend on the state at the previous time. The transition between control states depends on the policy pursued. In our example, this means that the sequence of fixation locations is determined by the policy. Each state gives rise to an outcome, o. We use the notation $\tilde{o}={\left[o_{1},o_{2},\ldots ,o_{T}\right]}^{T}$ to describe the sequence of outcomes through time. The outcome we are interested in here corresponds to “where I am looking.” Figure 2a shows graphically how one can factorize this kind of model.

The form of this generative model implies that we must perform a Bayesian model selection to decide on the best policy (sequence of saccades) to pursue. To do so, we define prior beliefs about the probability of each policy. Active inference mandates the selection of policies that minimize free energy. However, free energy is a function of outcomes, which are not known for future time steps. The solution to this is to select policies that minimize the expected free energy. To convert the expected free energy for each policy to a probability distribution (that sums to one), we use a softmax (normalized exponential) function, σ(·):

$$\begin{array}{ccc} P(\pi ) & = & \sigma \left(-\gamma \sum_{\tau >t}G(\pi ,\tau )\right),\\ G(\pi ,\tau ) & = & -E_{\tilde{Q}}\left[\ln\frac{P(o_{\tau },s_{\tau }|\pi )}{Q(s_{\tau }|\pi )}\right]\\ & = & D_{KL}\left[Q\left(o_{\tau }|\pi \right)\left|\right|P\left(o_{\tau }\right)\right]+E_{Q(s_{\tau }|\pi )}\left[H[P\left(o_{\tau }|s_{\tau }\right)]\right]. \end{array}$$

To account for future outcomes, we have used the (posterior predictive) distribution $\tilde{Q}\left(o_{\tau },s_{\tau }|\pi \right)=P\left(o_{\tau }|s_{\tau }\right)Q\left(s_{\tau }|\pi \right)$. The γ parameter is an inverse temperature parameter (associated with dopaminergic signaling) that weights how sensitive the prior is to differences in the expected free energy of policies. The above equation can be expressed in linear algebraic form as (Friston, FitzGerald, Rigoli, Schwartenbeck, & Pezzulo, 2017):

$$\begin{array}{ccc} \pi_{0} & = & \sigma \left(-\gamma \sum_{\tau >t}G_{\pi \tau }\right),\\ G_{\pi \tau } & = & o_{\pi \tau }\cdot \underset{\varsigma_{\pi \tau }}{\underbrace{\left(\ln o_{\pi \tau }-\ln C\right)}}+\;H\cdot s_{\pi \tau }. \end{array}$$

To perform inferences within these types of models, we apply equation 2.1, substituting in our discrete variables.

$$\begin{array}{ccc} & & Q\left(s_{\tau }|\pi \right)\propto \exp\left(E_{Q(s\backslash \tau |\pi )}\left[\ln P\left(\tilde{o},\tilde{s}|\pi \right)\right]\right)\\ & & \;=\exp\left(\ln P\left(o_{\tau }|s_{\tau }\right)+E_{Q}\left[\ln P\left(s_{\tau }|s_{\tau -1},\pi \right)\right]+E_{Q}\left[\ln P\left(s_{\tau +1}|s_{\tau },\pi \right)\right]\right). \end{array}$$

Expressing these distributions as vectors (and matrices) of their sufficient statistics, we can write

$$\ln s_{\pi \tau }=\ln A\cdot o_{\tau }+\ln B_{\pi \tau -1}s_{\pi \tau -1}+\ln B_{\pi \tau }\cdot s_{\pi \tau +1}.$$

It is then straightforward to construct a biologically plausible gradient ascent scheme with this solution as its fixed (attracting) point:

$$\begin{array}{ccc} s_{\pi \tau } & = & \sigma \left(v_{\pi \tau }\right),\;{\dot{v}}_{\pi \tau }=\epsilon_{\pi \tau },\\ \epsilon_{\pi \tau } & = & \ln A\cdot o_{\tau }+\ln B_{\pi \tau -1}s_{\pi \tau -1}+\ln B_{\pi \tau }\cdot s_{\pi \tau +1}-\ln s_{\pi \tau }. \end{array}$$

If we want to estimate a hidden state from this, we can use a Bayesian model average over policies. This gives $s_{\tau }=\sum_{\pi }\pi_{\pi }\cdot s_{\pi \tau }$ and completes our specification of the Bayesian belief updates required for categorical inference and decision making. For outcomes not yet observed, we can perform a Bayesian model average over $o_{\pi \tau }={\mathrm{As}}_{\pi \tau }$ to get a posterior predictive distribution, $o_{\tau }$. As we will see in section 2.3, this will act as our prior for a (lower-level) generative model of continuous states like forces and velocity.

### 2.3 Moving the Eyes

At the level of biological effectors (glands and muscles) and sensory receptors, the variables the nervous system must deal with are continuous. In other words, to enact a selected policy, it is necessary to map categorical representations to the physical world with continuous states and time. Muscles generate continuous forces, while photoreceptors signal light intensity. This means that there must be a generative model that maps the abstract, discrete outcomes from the generative (MDP) model above, to continuous data (Friston, Parr, et al., 2017).

Here, we consider how predictions about the next saccades are realized. An MDP outcome, representing fixation location, corresponds to one of several discrete saccadic targets, defined in continuous coordinates (v). If we associate each target location with the attracting (fixed) points of some continuous oculomotor dynamics, the prediction from the MDP effectively defines an equilibrium point that will attract the subsequent eye movement (cf. the equilibrium point hypothesis; Feldman, 2009). If there is some uncertainty about the particular location of the target, we can specify the predicted target location through a Bayesian model average of each location $\eta_{m}$ associated with a discrete outcome hypothesis or model, m:

$$\begin{array}{ccc} P(v|o) & = & N(\eta ,\Pi_{v}),\\ \eta & = & \sum_{m}o_{m}\cdot \eta_{m}. \end{array}$$

The ensuing location now plays the role of a latent or hidden cause that predicts the dynamics of continuous latent variables, $\dot{x}$ (eye position, angular velocity), and observable sensory data, y (vision and proprioception). The probability distributions that constitute the dynamic generative model are provided in Figure 2b. As in discrete state-space models, we use the tilde notation to indicate a trajectory. Here, however, the trajectory is represented as a vector of generalized motion (Friston, Trujillo-Barreto, & Daunizeau, 2008), $\tilde{x}={\left[x,x^{'},x^{''}\ldots \right]}^{T}$. We will use $x^{\left[i\right]}$ to refer to the ith order motion.

Using equation 2.1, but substituting latent variables x and v, we can write

$$\begin{array}{ccc} q(x^{\left[i\right]}) & \propto & \exp\left(E_{q\left(\tilde{v}\right)q\left(\tilde{x}\backslash i\right)}\left[\ln p\left(\tilde{y},\tilde{x},\tilde{v}\right)\right]\right),\\ q(v^{\left[i\right]}) & \propto & \exp\left(E_{q\left(\tilde{v}\backslash i\right)q\left(\tilde{x}\right)}\left[\ln p\left(\tilde{y},\tilde{x},\tilde{v}\right)\right]\right). \end{array}$$

Under the Laplace approximation (Friston, Mattout, Trujillo-Barreto, Ashburner, & Penny, 2007), the precision of these distributions depends only on the mean. This means we only need to optimize the mean explicitly. As this will be the maximum of the distribution, we can evaluate the above equations at their means and perform a generalized gradient ascent (Friston, Stephan, Li, & Daunizeau, 2010):

$$\begin{array}{ccc} {\dot{\mu }}_{x}^{\left[i\right]}-\mu_{x}^{\left[i+1\right]} & = & \frac{\partial }{\partial \mu_{x}^{\left[i\right]}}\ln p\left(y,{\tilde{\mu }}_{x},{\tilde{\mu }}_{v}\right),\\ {\dot{\mu }}_{v}^{\left[i\right]}-\mu_{v}^{\left[i+1\right]} & = & \frac{\partial }{\partial \mu_{v}^{\left[i\right]}}\ln p\left(y,{\tilde{\mu }}_{x},{\tilde{\mu }}_{v}\right). \end{array}$$

The expressions on the left ensure that when the gradient is zero, the motion of the mean is equal to the mean of the motion. We can supplement these equations, which mediate perceptual inference, with a differential equation equipping the system with low-level reflexes (i.e., the actions that cause contractions of the extraocular muscles) (Adams, Shipp, & Friston, 2013). These also minimize free energy but can be thought of as fulfilling predictions about sensory data by changing the external world:

$$\dot{a}=\frac{\partial }{\partial a}\ln p\left(y,{\tilde{\mu }}_{x},{\tilde{\mu }}_{v}\right)$$

**Figure 3:**
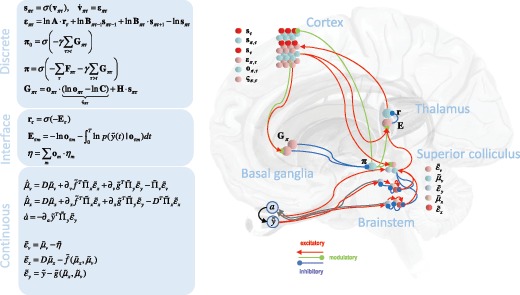
The anatomy of oculomotion. This schematic illustrates the dependencies between the variables in the equations described in the main text and summarized on the left. It does so in the form of a neural network with populations of neurons assigned to plausible anatomical locations. There is a remarkable degree of neuroanatomical plausibility to these assignments, including a common laminar origin for cortical projections to the striatum, superior colliculus, and higher-order thalamic nuclei. In addition, a dual cortico-subcortical input to the colliculus is necessitated by this scheme, as are the excitatory-inhibitory connections of the direct pathway through the basal ganglia. The equations in the box on the top left describe variational message passing in a Markov decision process. The bottom box gives the Bayesian filtering equations of the sort usually associated with predictive coding. The middle box expresses the descending messages derived from Bayesian model averaging and the ascending messages that result from model reduction. We have not shown the neuronal representation of the C matrix, encoding preferences. In previous papers, we show that this is likely to be represented in the mapping from higher cortical areas (Friston, Rosch, et al., 2017), such as the dorsolateral prefrontal cortex, an area that houses representations that endure over a longer temporal scale (Parr & Friston, 2017d) and connects to the frontal eye fields.

Figure 3 (left lower panel) shows these equations for the generative model in Figure 2b.

### 2.4 Ascending Messages

To recap, we have specified both discrete and continuous state-space models. We have shown how predictions of the former model can play the role of (empirical) prior beliefs in the latter. The final thing to specify is the process by which the continuous state-space model informs the discrete model. In other words, what sort of evidence is passed from the continuous to the discrete part of the (active) inference scheme? In brief, the discrete part of the generative model provides prior constraints on the continuous part, while the continuous part reciprocates with Bayesian model evidence for the discrete hypotheses entertained by the discrete part to enable Bayesian belief updating. This updating entails the selection of alternative hypotheses (outcomes) that constitute empirical priors at the continuous level.

To adjudicate between these hypotheses, we need to compute the posterior probabilities over each outcome (e.g., target fixation). We give an abbreviated outline of this (Bayesian model reduction) procedure here (for a more technical account, see Friston, Litvak, et al., 2016). Given that the only differences between discrete hypotheses are their priors, we can use Bayes' rule to write the posterior odds ratio between the full and reduced model, cancelling the likelihood terms:

$$\frac{p\left(\tilde{v}|\tilde{y},o_{m}\right)p\left(\tilde{y}|o_{m}\right)}{p\left(\tilde{v}|\tilde{y},o\right)p\left(\tilde{y}|o\right)}=\frac{p(\tilde{v}|o_{m})}{p(\tilde{v}|o)}$$

We can replace the posteriors here with those that we compute using the Bayesian filtering approach outlined above. Rearranging this, we get

$$p\left(\tilde{y}|o_{m}\right)q\left(\tilde{v}|o_{m}\right)=\frac{p(\tilde{v}|o_{m})}{p(\tilde{v}|o)}q\left(\tilde{v}|o\right)p\left(\tilde{y}|o\right).$$

Integrating both sides with respect to the hidden cause gives

$$p\left(\tilde{y}|o_{m}\right)=p\left(\tilde{y}|o\right)\int \frac{p(\tilde{v}|o_{m})}{p(\tilde{v}|o)}q\left(\tilde{v}|o\right)d\tilde{v}.$$

As the free energy approximates the negative log evidence, this can be rewritten as

$$F\left(o_{m}\right)=F\left(o\right)-\ln\int \frac{p(\tilde{v}|o_{m})}{p(\tilde{v}|o)}q\left(\tilde{v}|o\right)d\tilde{v}$$

Notably, this means that the free energy of any hypothesis, m, can be calculated from the free energy of the full outcome model without having to explicitly compute the posteriors associated with the latent variables in m. This is a slightly technical point that from a computational perspective, affords a very simple and efficient form of Bayesian model comparison. In other words, the evidence for different hypotheses or models at the discrete level can be computed directly and easily from the sufficient statistics of posterior beliefs encoded at the continuous level. In terms of neurobiology, this speaks to the biological possibility of belief propagation from the continuous to the discrete domains.

To convert the evidence for each model back to discrete time, we integrate the model evidence (free energy) over the time period corresponding to one theta cycle. This is then combined with the prior over the model to give a vector (***E***) that can be passed through a softmax function to give the posterior over each outcome model, $r_{\tau }$ (Friston, Parr, et al., 2017). This plays the role of a discrete observation or outcome from the point of view of the MDP (see Figure 3).

This concludes our technical description of belief propagation between discrete and continuous parts of a generative model. A worked example of how this sort of belief propagation of message passing could work in the brain is provided in the final section (using the update equations in Figure 3). To motivate the interpretation of these simulations, we now consider the basic neurobiology of the oculomotor system and how its computational architecture could support belief propagation of this sort.

## 3 The Computational Anatomy of Oculomotion

In section 2, we described the problem the brain faces in making discrete decisions about where to look and the continuous inferences required to realize and update these decisions. We outlined the computations mandated by active inference in solving these problems, with a special focus on the message passing between discrete and dynamic domains. In this section, we associate these computations with their neurobiological substrates. While this assignment is speculative, it is constrained by both the anatomy of message passing and the presence (or absence) of connections in the brain. Figure 3 shows the consistency between the computational anatomy of oculomotion and the networks known to support oculomotor function. In the following, we describe the cortical, subcortical, and brain stem components of this network (Parr & Friston, 2017a). This section concludes with an analysis of the superior colliculus, a structure uniquely placed to translate discrete decisions into target locations in a continuous state space.

### 3.1 Cerebral Cortex

The cerebral cortex is a laminar structure, with layer-specific projections and terminations (Felleman & Van Essen, 1991). The connectivity implied by inference using a Markov decision process closely resembles this pattern (Friston, Rosch, et al., 2017). Specifically, the inference scheme we have described involves several distinct types of variables that receive messages from a subset of the other variables. This implies a stereotyped pattern of connectivity between these groups (or layers) of computational units. Consistent with cortical laminae, external input targets only one layer. Outputs of different types arise from defined populations. In this section, we use known neuroanatomy to constrain the assignment of computational units to their appropriate laminae.

Layer IV of the cortex receives ascending connections from lower areas (Shipp, 2007), or from first-order thalamic nuclei. The computational units that receive this input are the error units, $\epsilon_{\pi \tau }$, suggesting that these occupy this layer. This also implies that $r_{\tau }$, the subcortical projection to layer IV, is likely to be represented by neurons in first-order thalamic relay nuclei, such as the lateral geniculate nucleus (Herkenham, 1980). Layer III gives rise to ascending connections. These are not shown here but would arise from neurons encoding the state $s_{\tau }$ at that hierarchical level (Friston, Rosch, et al., 2017). For simplicity, we consider a single cortical area, the frontal eye field, omitting the parietal (Corbetta et al., 1998; Gaymard, Lynch, Ploner, Condy, & Rivaud-Péchoux, 2003; Parr & Friston, 2017b; Shipp, 2004) and occipital (Bruce & Tsotsos, 2009) contributions to this system.

Layer V of the cortex has several subcortical targets (Kasper, Larkman, Lübke, & Blakemore, 1994; Ojima, Murakami, & Kishi, 1996). It is the layer that houses the pyramidal cells of Betz in the motor cortex that project to lower motor neurons in the spinal cord. In addition, layer V gives rise to projections to the second-order thalamic nuclei, such as the pulvinar, the superior colliculus, and the basal ganglia (Fries, 1985; Hübener & Bolz, 1988; Shipp, 2007). As Figure 3 shows, units encoding $o_{\pi \tau }$ send messages to all of these anatomical homologues. They participate in the evaluation of the expected free energy in the striatum, the model averaging of continuous time models by the superior colliculus, and Bayesian model reduction by the thalamus. That the $E_{\tau }$ units of the thalamus receive cortical projections from layer V suggests that these neurons must be located in second-order thalamic nuclei (Crick & Koch, 1998; Rockland, 1998; Sherman, 2007).

### 3.2 Basal Ganglia

As noted above, the basal ganglia receive input from cortical layer V, encoding predictions about discrete outcomes. This input, in addition to a signal from the error units, $\varsigma_{\pi \tau }$, is used to compute the expected free energy, $G_{\pi }$, of each policy. The basal ganglia are well recognized to be involved in policy evaluation (Gurney, Prescott, & Redgrave, 2001; Jahanshahi, Obeso, Rothwell, & Obeso, 2015). Most of the cortical inputs to the basal ganglia target the striatum (Alexander & Crutcher, 1990; Shipp, 2017), implying the expected free energy is represented by medium spiny neurons in this structure. These give rise to inhibitory GABAergic projections to the substantia nigra pars reticulata, which itself projects to the superior colliculus (Hikosaka & Wurtz, 1983). This direct pathway connectivity is remarkably consistent with the influence of $G_{\pi }$ on π and π on ${\tilde{\epsilon }}_{v}$. The latter influence is in the Bayesian model averaging over expected outcomes to generate a prior mean, $\tilde{\eta }$, for the implementation of the policy in continuous time. The output nuclei of the basal ganglia participate in an additional Bayesian model averaging of hidden states. This is mediated by modulatory projections (via thalamic relays) to superficial layers of the cortex (Haber & Calzavara, 2009; McFarland & Haber, 2002).

### 3.3 The Brain Stem

The brain stem is the source of the cranial nerves to the extraocular muscles. This suggests that brain stem structures engage in continuous message passing. We have previously demonstrated that the anatomy of this message passing is not only consistent with the connectivity of the brain stem, but also that it reproduces electrophysiological responses in these structures, and the same deficits as in neurological patients when lesioned (Parr & Friston, 2018). In addition, these nerves carry proprioceptive information from the muscles (Cooper & Daniel, 1949; Cooper, Daniel, & Whitteridge, 1951), while the midbrain receives optic nerve fibers from the retinotectal pathway (Linden & Perry, 1983). Given beliefs about the current position and velocity of the eyes, ${\tilde{\mu }}_{x}$, it is possible to make predictions about the resulting sensory input (Adams, Perrinet, & Friston, 2012; Friston, Adams, Perrinet, & Breakspear, 2012; Perrinet, Adams, & Friston, 2014). This induces a sensory prediction error ${\tilde{\epsilon }}_{y}$ that is minimized by action. This implies that the midbrain and pontine nuclei responsible for signals to the extraocular muscles must contain neurons that broadcast these errors. As the brain stem nuclei form the nodes of the network engaged in continuous inference, they must receive input from the region mapping decisions into this space. The obvious candidate for this region is the superior colliculus.

### 3.4 The Superior Colliculus

**Figure 4:**
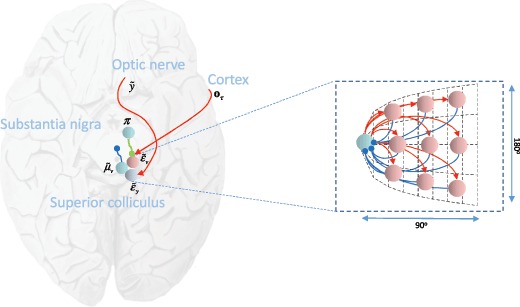
The discrete-continuous interface. This schematic shows the connectivity between the neuronal populations in the superior colliculus in greater detail. The transverse section through the midbrain allows us to depict the terminations in the optic tectum from the optic nerve. We also illustrate the topographical arrangement of the fixation (rostral pole) and buildup (distributed throughout) cells, and the connectivity between these implied by our formulation. Note that burst neurons are the most dorsal, with buildup neurons found more ventrally. As the schematic shows, this would be consistent with the proposed extrinsic (between regions) connectivity, as each population is oriented toward the regions it is connected to. The intrinsic (within region) connectivity between buildup and fixation neurons is shown on the right, conforming to the known retinotopy of the colliculus (Paré, Crommelinck, & Guitton, 1994; Quaia, Aizawa, Optican, & Wurtz, 1998). The angles indicate the coordinates of the visual field represented at each point in the colliculus.

The superior colliculus is the interface between the forebrain and brain stem networks. It is the recipient of cortical (Hanes & Wurtz, 2001) and basal ganglia projections (Hikosaka & Wurtz, 1983; see Figure 4) and is intimately connected to the oculomotor system within the brain stem (Sparks, 2002). As such, it sits at the anatomical boundary between the discrete and continuous networks. It is found in the dorsal midbrain, at the same level as the oculomotor nucleus. Like the cortex, it is a laminar structure, with different electrophysiological responses in different subsets of cells. There are three broad groups of these neurons, as illustrated in Figure 4. These are the burst, fixation, and buildup cells (Ma, Graybiel, & Wurtz, 1991; Munoz & Wurtz, 1995a).

We have previously argued (Parr & Friston, 2018) that these groups correspond to three different types of computational unit. Burst cells, which fire at the start of a saccade, have the properties we would expect from neurons signaling visual prediction error, ${\tilde{\epsilon }}_{y}$. This is consistent with the fact that a subset of retinal ganglion cells synapses within the colliculus and that some collicular cells respond to visual stimuli (Mays & Sparks, 1980; Wurtz & Mohler, 1976). Fixation neurons are active during fixations, and we have associated these with the expectation neurons encoding target fixation locations, ${\tilde{\mu }}_{v}$. Consistent with the computational anatomy of Figures 3 and 4, it is this group that projects to the brain stem centers for saccade generation (Gandhi & Keller, 1997).

Buildup neurons show a pattern of activation consistent with a population encoding (Anderson, Keller, Gandhi, & Das, 1998; Lee, Rohrer, & Sparks, 1988). A traveling hill of excitation moves from a peripheral location toward the rostral pole of the colliculus during a saccade (Munoz & Wurtz, 1995b). At a population level, these neurons can be thought of as expressing a prediction error between a target fixation and the current eye position, ${\tilde{\epsilon }}_{v}$ (Sparks, 1986). The movement toward the pole, representing the foveal location, can be thought of as encoding the reduction in prediction error as the eye moves closer to its target. That this occurs at the population level suggests that buildup neurons individually code for discrete spatial regions.

The discretized encoding of continuous variables by these units is consistent with their computational role, evaluating the difference between $\tilde{\eta }$, parameterizing competing hypothetical models, and the estimated position in continuous coordinates, ${\tilde{\mu }}_{v}$. Specifically, each neuron may encode the prediction error associated with a particular hypothesis, ${\tilde{\eta }}_{m}$, with activity weighted by the prior probability of that hypothesis $o_{m}$. The conversion from discrete to continuous coordinates then simply requires that the connection strengths between these neurons and the fixation neurons at the pole vary with distance. The inhibitory connections (Munoz & Istvan, 1998) from buildup to fixation neuron should be stronger if the anatomical distance between the two is greater. In summary, electrophysiological properties corroborate the neuroanatomical evidence that the superior colliculus is the discrete-continuous interface of the oculomotor system, and the topography of buildup and fixation neurons hints at the computational mechanisms that map between them. We next use the neurobiological pointers established in this section to interpret simulated oculomotor control in terms of established electrophysiological responses in the oculomotor system.

## 4 Simulations

Our aim in performing these (minimal) simulations is to illustrate the interactions between the discrete and continuous domains of the oculomotor system. As such, we chose a simple behavioral paradigm with three possible fixation locations (left, right, and center). We then set the prior preferences (through the C matrix) so that proprioceptive data are preferred that are consistent with central fixation initially, then with leftward fixation, rightward fixation, and finally central fixation again. This is consistent with the instruction to look at a sequence of targets at each of these locations. The structure of the model we used is as depicted in Figure 1. The continuous part employs the belief that the eyes are drawn toward an attracting location, and this is implemented by an action that has the effect of a Newtonian torque. The details of this can be found in Parr and Friston (2018) and are very closely related to those in previous formulations of oculomotion (Adams et al., 2012; Friston, Adams, et al., 2012; McSpadden, 1998; Perrinet et al., 2014). The discrete part is formally similar to that used in Mirza et al., 2016 and Parr and Friston (2017b, 2017c).

**Figure 5:**
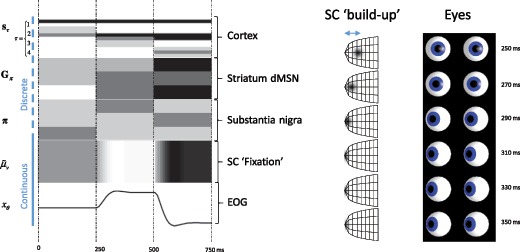
Simulated electrophysiological responses. This figure shows the electrophysiological responses we would expect to observe under the process theory associated with active inference. On the left, we show neuronal firing rates (representing approximate posterior beliefs), depicted in the form of a raster plot. These are synchronized across all of the neurons shown. Darker colors indicate a greater firing rate. In the cortex (frontal eye field), we show neurons representing the possible fixation targets. The first three rows indicate the neurons representing left, center, and right at the first discrete time step (τ=1). These three options are then replicated in the next three rows, but reporting the second time step. The third and fourth steps are similarly represented. In the striatum, direct pathway medium spiny neurons (dMSN) represent the expected free energy of each of the three policy options (saccade right, saccade center, and saccade left). These then inhibit their corresponding neurons in the substantia nigra pars reticulata that represent the posterior beliefs about these policies. Note the inversion of striatal activity in the substantia nigra as a result of this inhibition. We show the activity of a superior collicular (SC) fixation neuron to illustrate neuronal firing representing a continuous variable, with the horizontal electro-oculography trace below to depict the movement of the eyes. The right panel shows simulated activity across the superior collicular buildup layer during a saccade, with the corresponding eye positions. Population activity is depicted in terms of a gaussian intensity where the distance between the mean and the collicular pole is equal to the prediction error (as in Parr & Friston, 2018).

Figure 5 shows the results of simulating active vision in this model. Crucially, this type of simulation allows us to show what is happening at different neuroanatomical locations simultaneously and gives a sense of the functional interaction of these areas. The results are presented in the form of raster plots, as if we had recorded from single neurons in each of the simulated brain areas. Representations in the cortex and basal ganglia update on a theta cycle (4 Hz), while superior colliculus buildup cells translate this into continuous time for collicular fixation neurons. These induce (via brain stem circuits) changes in eye position, as shown in the simulated electro-oculography trace. Note that these neurons vary continuously with eye position, unlike the neurons in the discrete compartments, which encode the probability of an alternative fixation point.

Intuitively, we can see how the mapping from discrete to continuous occurs. At 250 ms, the cortex updates its representations. These cause the expected free energy under each policy to change, inducing updates in the striatum. Through the direct pathway, this causes inhibition in the substantia nigra pars reticulata, resulting in the selection of a new policy (“look right”). A Bayesian model average over outcomes then results in the selection of superior collicular buildup neurons that represent the error between the current belief about eye position (given by the fixation neurons) and the anticipated rightward location. These then induce changes in the fixation neuron activity. During the next 100 ms, as the eyes move to fulfill this belief, we can see the resolution of the prediction error in the superior colliculus buildup layer through the movement of the hill of activity toward the pole.

## 5 Discussion

### 5.1 Message Passing and Inference

Variational message passing is not the only local message passing scheme that has been associated with neuronal signaling. Belief propagation (Pearl, 2014) is one alternative. The message passing under this approach is perfectly consistent with the free energy principle, as the requisite messages can be shown to minimize the Bethe free energy (Yedidia, Freeman, & Weiss, 2005). However, although attempts have been made to link belief propagation to neural signaling (Jardri & Denève, 2013), the resulting architectures turn out to be more complicated than the equivalent variational schemes. The primary reason for this is the recursive computation of such messages. Each message is derived from another message rather than from posterior beliefs about each variable. Variational messages passed from one population of neurons are derived from the posterior beliefs represented by these neurons, mapped through a set of synaptic weights. This means that the same set of neurons can be used to compute multiple different messages. In contrast, a recursive scheme requires streams of forward and backward messages that do not directly interact.

A further possibility is that the messages we have described might be computed through sampling, or Monte Carlo, approaches (Hastings, 1970). One argument against this is that these methods tend to take much longer to converge. In a biological setting (requiring online inference), this means that animals would spend more time in surprising states (associated with a lower model evidence). In short, while Monte Carlo approaches may achieve a greater accuracy, they induce a larger (path integral of the) free energy than variational approaches.

### 5.2 Physiology and Behavior

The approach we have described here is capable of reproducing a wide range of physiological and behavioral phenomena in the oculomotor system and more generally. In the context of the oculomotor system, we have previously shown that the signals we have simulated bear a close resemblance to those measured in brain stem nuclei (Parr & Friston, 2018). Most striking, we found that simulated collicular buildup neuron responses qualitatively reproduced single-unit recordings published in the experimental literature (Munoz & Wurtz, 1995b).

Lesions to these models induce similar behavioral syndromes to those found in neurological patients with damage to the associated neuroanatomy. By disrupting neuronal message passing (i.e. inducing disconnection syndromes; Geschwind, 1965), we have simulated visual neglect (Parr & Friston, 2017b) and internuclear ophthalmoplegia (Parr & Friston, 2018). The white matter disconnections associated with these syndromes are the superior longitudinal fasciculus (Doricchi & Tomaiuolo, 2003) and the medial longitudinal fasciculus (Virgo & Plant, 2017), respectively. The locations of these synthetic lesions constrain the computational anatomy, and their nature endorses the notion that the brain engages in variational inference.

More generally, models based on active inference have a high degree of face validity, in that they reproduce a wide range of neurobiological phenomena. These range from single cell responses, including place fields (Friston, FitzGerald, Rigoli, Schwartenbeck, & Pezzulo, 2017) and midbrain dopamine activity (Friston et al., 2014), to evoked responses, including those associated with classic working memory tasks (Parr & Friston, 2017d). They have been used to generate behaviors as diverse as exploration (Friston et al., 2015; Mirza et al., 2016), handwriting (Friston, Mattout, & Kilner, 2011), eye-blink conditioning (Friston & Herreros, 2016), habit formation (FitzGerald, Dolan, & Friston, 2014), communication (Friston & Frith, 2015), and insight (Friston, Lin, et al., 2017). In addition to these theoretical accounts, active inference has been used pragmatically to model behavior and to characterize individuals according to the parameters of their prior beliefs (Adams, Bauer, Pinotsis, & Friston, 2016; Mirza, Adams, Mathys, & Friston, 2018; Schwartenbeck & Friston, 2016).

### 5.3 Generalizations

The issues described in this article generalize beyond eye movements. Any neurobiological system that needs to make decisions and implement these via some physical effector must solve the problem we have described here. This is vital for (but not exclusive to) speech, locomotion, and autonomic regulation. Language is made up of discrete units (phonemes, words, sentences) that are expressed as continuous changes in auditory frequencies generated by contraction of the laryngeal (and pharyngeal) muscles (Simonyan & Horwitz, 2011). Walking involves taking a series of discrete steps, each of which requires a careful coordination of skeletal muscles (Ijspeert, 2008; Winter, 1984). Interoceptive states are frequently divided into discrete dichotomies including fed versus fasting (Kalsbeek, la Fleur, & Fliers, 2014; McLaughlin & McKie, 2016; Roh, Song, & Kim, 2016), diastole versus systole, and sympathetic versus parasympathetic (McDougall, Münzberg, Derbenev, & Zsombok, 2014; Owens, Friston, Low, Mathias, & Critchley, 2018). Each of these induces continuous changes in enzyme activity, blood pressure, or smooth muscle contractions. The form of the variational message passing will be very similar for each of these processes, but the variables represented will differ. This suggests a similar pattern of cortico-subcortical connectivity, but differing regions of cortex, and different subcortical components.

In this article, we have chosen to focus on a fairly concrete problem: deciding where to look and how to do this. For more abstract decisions, perhaps at higher hierarchical levels in the brain (Badre, 2008; Badre & D'Esposito, 2009; Christoff, Keramatian, Gordon, Smith, & Mädler, 2009; Rasmussen, 1985), it may be necessary to integrate beliefs across multiple modalities. A challenge for future work is to incorporate the set of beliefs that constitute an emotional state, as emotions are often thought to contribute to “irrational” behaviors. It is not always easy to intuit how such behaviors might be Bayes optimal.

One line of research into these issues frames them as questions about interoceptive inference (Ondobaka, Kilner, & Friston, 2017; Seth, 2013; Seth & Friston, 2016). Given beliefs about (abstract) variables that have both interoceptive and exteroceptive sensory consequences, it becomes clear that policies must minimize expected free energy in both domains. For example, a belief that a predatory animal is present implies that the sympathetic nervous system should be active, but also that visual data are consistent with the presence of said animal. Anatomically, these dependencies are consistent with the sensory and autonomic targets of the amygdala (LeDoux, Iwata, Cicchetti, & Reis, 1988; Ressler, 2010). A tachycardia then carries (weak) evidence for the presence of a scary animal and could influence policy selection even in the absence of exteroceptive evidence. This suggests a framework in which an emotional state may influence decision making in an apparently irrational way that is entirely compatible with the formulation we have described here.

### 5.4 Future Directions

We hope to further the ideas in this work both theoretically and empirically. Two important theoretical issues need to be addressed in greater depth than we have space for here. The first is a thorough comparison of alternative message-passing schemes in relation to their anatomical and physiological plausibility. The second is a generalization of the inferences required for oculomotor decisions (and their motoric implementation) to other systems. While the ideas we have presented are generally applicable, it will be necessary to specify the generative models required to solve locomotive, autonomic, and abstract decision-making problems. Finally, although the computational anatomy we have proposed has a high degree of face validity, it will be necessary to establish its predictive validity.

One way to do so would be to use computational fMRI (Schwartenbeck, FitzGerald, Mathys, Dolan, & Friston, 2015), fitting this model to oculomotor behavior, and looking for brain regions that show activity patterns consistent with the simulated neuronal responses. We hypothesize that these regions will match the computational anatomy illustrated in Figure 3. An alternative would be to use single-unit responses from each brain area recorded during an oculomotor task. One could then compare each simulated neuronal response to each recording and construct a confusion matrix of the evidence for each synthetic signal in each region. We would expect a greater evidence for each signal that we have associated with each region above.

## 6 Conclusion

In this article, we have described the discrete and continuous message passing that must be performed in an oculomotor system that realizes a sequence of saccadic fixations. We have illustrated the remarkable consistency between the belief propagation or message passing implied by active inference and the anatomy of the oculomotor system. This accounts for several neuroanatomical observations, including the dual input from frontal eye fields and the substantia nigra to the superior colliculus, and the common laminar origin of axons that target the striatum, second-order thalamus, and midbrain tectum. Finally, we simulated electrophysiological responses as saccadic targets are selected and as the eyes move to implement that saccade. This shows, functionally, how the superior colliculus is uniquely positioned to act as the interface between the discrete and continuous oculomotor systems.

## Software Note



Although the generative model changes from application to application, the belief updates described in this article are generic and can be implemented using standard routines (here spm_MDP_VB_X.m). These routines are available as Matlab code in the SPM academic software: http://www.fil.ion.ucl.ac.uk/spm/. Simulations of the sort reported above can be reproduced (and customized) via a graphical user interface by typing in >> DEM and selecting the ‘visual foraging’ demo.
